# Perioperative Opioid Exposure in Adolescents and Long-Term Psychiatric/Utilization Outcomes: A Propensity-Matched Real World Cohort Study

**DOI:** 10.1192/j.eurpsy.2026.11476

**Published:** 2026-07-31

**Authors:** A. S. Verma, V. Sharma, A. Pathak, R. Gupta

**Affiliations:** 1 Psychiatry, Case Western Reserve University/ Metrohealth, Cleveland, United States; 2 Psychiatry, Dayanand Medical College & Hospital, Ludhiana, India; 3 Psychiatry, Cleveland Clinic Main Campus, Cleveland; 4 Psychiatry, Brigham & Women’s Faulkner Hospital; 5 Psychiatry, Harvard Medical School, Boston, United States

## Abstract

**Introduction:**

Use of opioids peri-operatively, for both analgesia and anesthesia, is being highlighted negatively due to the opioid crisis in the United States. There is evidence that being exposed to opioids peri-operatively, alters neurobiology by disrupting the dopamine and serotonin pathways, through action on the hypothalamic-pituitary-axis, especially in the pliable adolescent brain. This can result in development of long term psychiatric comorbidity, opioid use disorder and consequent increased emergency healthcare visits.

**Objectives:**

To test whether perioperative opioid exposure after common adolescent procedures is associated with long-term psychiatric diagnoses, emergency department (ED) utilization, overdose, and indicators of opioid use treatment, when compared with non-opioid analgesia.

**Methods:**

We conducted a retrospective cohort study on the TriNetX US Collaborative Network (71 health systems). Adolescents **aged 12–19** undergoing common procedures (appendectomy, tonsil/adenoid, select orthopedic and dental procedures) were identified. The opioid cohort had a perioperative opioid use (−3 to +7 days around surgery); the comparison cohort had no perioperative opioid use. Key **exclusions at baseline:** prior opioid-related disorders (F11), any substance-use disorders (F10–F19), and chronic pain (G89.2). Outcomes (depression, anxiety, PTSD, sleep disorders, suicidal ideation/attempt, ED utilization, opioid poisoning/overdose, long-term opioid therapy [Z79.891], and MOUD/overdose-related medications [buprenorphine, methadone, naltrexone, naloxone]) were assessed from day 1 to 3,650 (~10 years) post-index. Propensity score matching 1:1 was performed on age, sex, and ethnicity; measures of association and Kaplan–Meier analyses were run on matched cohorts (Table 1).

**Results:**

After matching (n=**77,810** per cohort), perioperative opioids were associated with higher risk of depression (RR 1.195, 95%CI 1.126–1.268), anxiety (RR 1.215, 1.168–1.264), sleep disorders (RR 1.296, 1.238–1.356), suicidal ideation/attempt (RR 1.121, 1.027–1.223), ED utilization (RR 1.187, 1.158–1.215), and opioid poisoning/overdose (RR 1.522, 1.049–2.208). Long-term opioid therapy (Z79.891) was not increased (RR 0.952, 0.820–1.104). Initiation of MOUD/overdose-related medications was markedly higher (RR 2.912, 2.572–3.298). Notably, PTSD diagnoses were lower in the opioid group (RR 0.581, 0.554–0.608). Kaplan–Meier results were directionally consistent for most outcomes (Table 2)

**Image 1:**
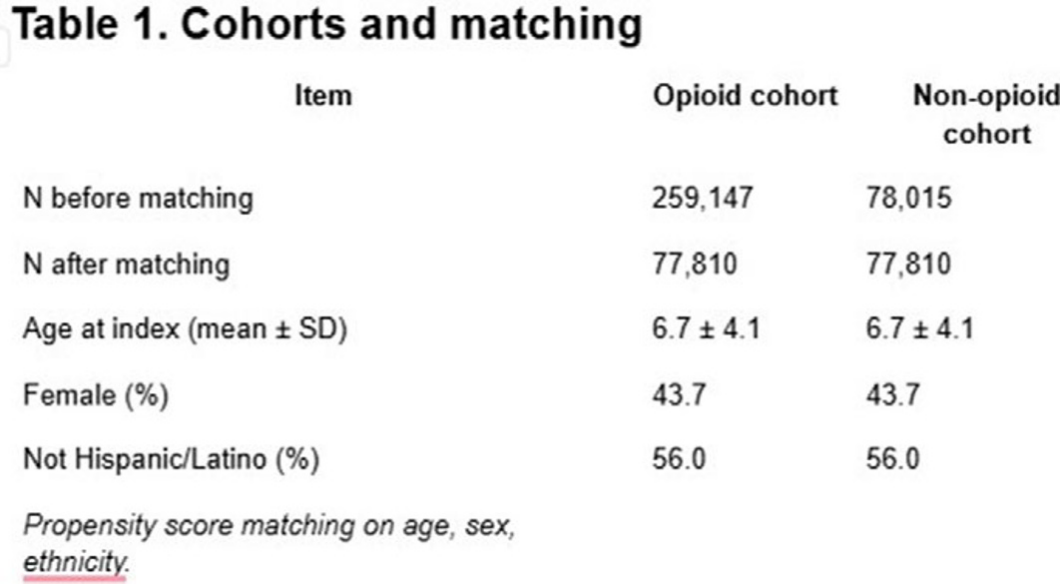
Image 1: Long description.

**Image 2:**
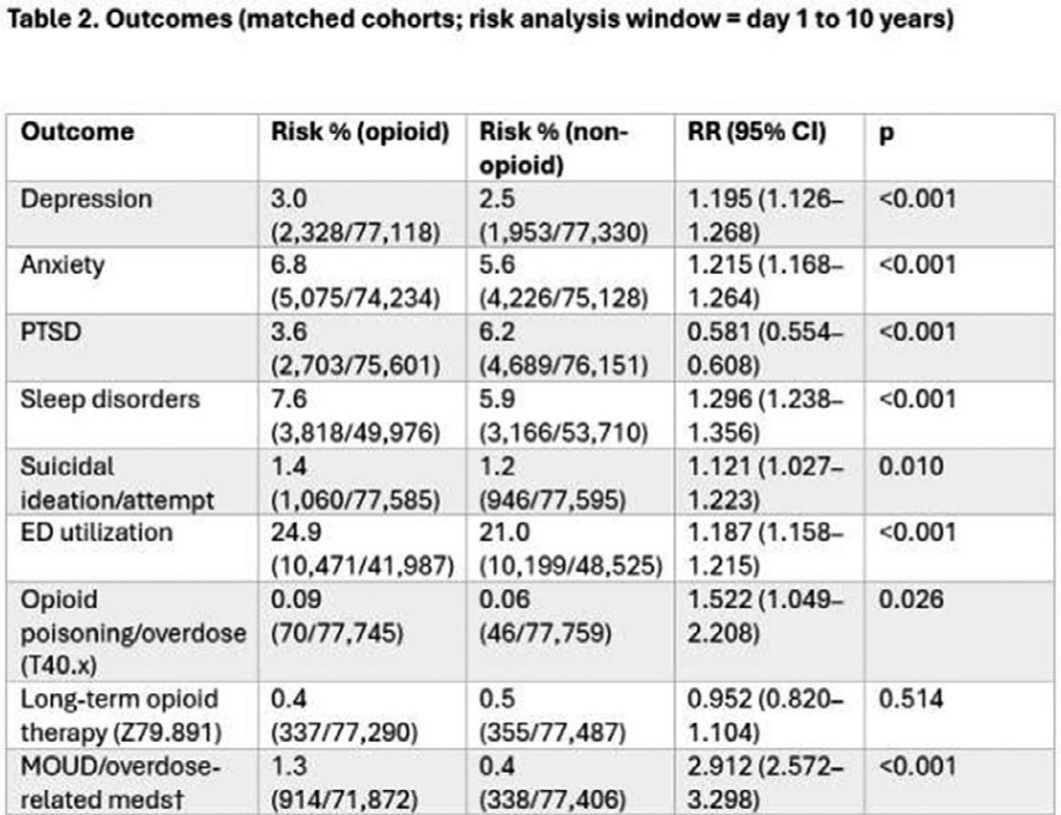
Image 2: Long description.

**Conclusions:**

In adolescents undergoing common procedures, perioperative opioid exposure was associated with small-to-moderate increases in long-term depression, anxiety, sleep disorders, suicidal ideation/attempt, ED utilization, and overdose, and a large increase in MOUD/overdose-related medication exposure. Findings support minimizing perioperative opioid exposure and proactive mental-health surveillance after surgery.

**Disclosure of Interest:**

None Declared

## Long descriptions

### Image 1: Long description

The table contains three columns: Item, Opioid cohort, and Non-opioid cohort.

* N before matching: 259,147 for Opioid cohort and 78,015 for Non-opioid cohort.

* N after matching: 77,810 for both cohorts.

* Age at index (mean plus or minus S D): 6.7 plus or minus 4.1 for both cohorts.

* Female (%): 43.7 for both cohorts.

* Not Hispanic/Latino (%): 56.0 for both cohorts.

A footer note states: Propensity score matching on age, sex, ethnicity.

Navigate back to Image 1:.

### Image 2: Long description

The table contains five columns: Outcome, Risk % (opioid), Risk % (non-opioid), R R (95% C I), and p.

* Depression: Opioid risk 3.0 (2,328/77,118); Non-opioid risk 2.5 (1,953/77,330); R R 1.195 (1.126–1.268); p <0.001.

* Anxiety: Opioid risk 6.8 (5,075/74,234); Non-opioid risk 5.6 (4,226/75,128); R R 1.215 (1.168–1.264); p <0.001.

* P T S D: Opioid risk 3.6 (2,703/75,601); Non-opioid risk 6.2 (4,689/76,151); R R 0.581 (0.554–0.608); p <0.001.

* Sleep disorders: Opioid risk 7.6 (3,818/49,976); Non-opioid risk 5.9 (3,166/53,710); R R 1.296 (1.238–1.356); p <0.001.

* Suicidal ideation/attempt: Opioid risk 1.4 (1,060/77,585); Non-opioid risk 1.2 (946/77,595); R R 1.121 (1.027–1.223); p 0.010.

* E D utilization: Opioid risk 24.9 (10,471/41,987); Non-opioid risk 21.0 (10,199/48,525); R R 1.187 (1.158–1.215); p <0.001.

* Opioid poisoning/overdose (T40.x): Opioid risk 0.09 (70/77,745); Non-opioid risk 0.06 (46/77,759); R R 1.522 (1.049–2.208); p 0.026.

* Long-term opioid therapy (Z79.891): Opioid risk 0.4 (337/77,290); Non-opioid risk 0.5 (355/77,487); R R 0.952 (0.820–1.104); p 0.514.

* M O U D/overdose-related medst: Opioid risk 1.3 (914/71,872); Non-opioid risk 0.4 (338/77,406); R R 2.912 (2.572–3.298); p <0.001.

Navigate back to Image 2:.

